# So similar, yet so different: α-synuclein recognition of the isoform-specific VDAC protein–lipid interface

**DOI:** 10.64898/2026.10.13.758914

**Published:** 2026-10-19

**Authors:** Tatiana K. Rostovtseva, Megha Rajendran, William M. Rosencrans, María Queralt-Martín, Mya S. Wolfe, Nina A. Bautista, Bethel G. Beyene, Motahareh G. Larimi, Alexander J. Sodt, Sergey M. Bezrukov

## Abstract

In mammals, VDAC exists as three isoforms – VDAC1, VDAC2, and VDAC3 – each characterized by a distinct tissue-dependent distribution and physiologic role. Isoform-specific knockout studies in cells and mice demonstrate distinct physiological roles for each VDAC isoform. The isoforms share about 70% sequence homology and a conserved β-barrel structure and, when reconstituted into planar lipid membranes, form similar highly conductive, anion-selective, voltage-gated channels. A natural question, then, is how relatively minor differences in isoform sequences lead to striking differences in their interactions with protein partners and, consequently, in their physiological functions. To address this question, we used a small, intrinsically disordered cytosolic protein α-synuclein (αSyn) as a sensitive molecular probe. A comparative study with recombinant VDACs reconstituted into planar lipid membranes shows that, depending on the isoform, the αSyn-induced blockage exhibits up to 100-fold differences in on-rates and equilibrium constants. The isoforms also display distinct translocation regimes for αSyn, as evidenced by altered dependences of the off-rates on the applied voltage. Deletion of cysteine residues has a significant effect on αSyn binding. Combined with corresponding data obtained for VDAC2 substates – which show an order of magnitude variation in on-rates while maintaining the conserved equilibrium constants – and the results of molecular dynamics simulations, these findings point to a previously unrecognized step in VDAC inhibition by αSyn: an isoform-specific αSyn interaction with the VDAC protein-lipid interface. This finding opens a new paradigm for understanding VDAC isoform functions and developing isoform-specific therapeutics.

**New & Noteworthy:**

Comparative analysis of α-synuclein interaction with three mammalian VDAC isoforms, complemented by corresponding data for VDAC2 substates and results from molecular dynamics simulations, reveals a new step in the chain of events leading to VDAC blockage. Specifically, our findings point to the isoform-specific interaction of αSyn with VDAC protein-lipid interface, thereby guiding a new strategy in the search for VDAC-targeting drugs.

